# Effect of Cilastatin on Cisplatin-Induced Nephrotoxicity in Patients Undergoing Hyperthermic Intraperitoneal Chemotherapy

**DOI:** 10.3390/ijms22031239

**Published:** 2021-01-27

**Authors:** Matilde Zaballos, Mercedes Power, María Iluminada Canal-Alonso, María Ángeles González-Nicolás, Wenceslao Vasquez-Jimenez, Pablo Lozano-Lominchar, Pilar Cabrerizo-Torrente, Natividad Palencia-García, Susana Gago-Quiroga, María Dolores Ginel-Feito, Consuelo Jiménez, Alberto Lázaro, Luis González-Bayón

**Affiliations:** 1 Department of Anesthesiology and Reanimation, Hospital General Universitario Gregorio Marañón, 28040 Madrid, Spain; mati@plagaro.net (M.Z.); poweresteban@gmail.com (M.P.); marilucanal@gmail.com (M.I.C.-A.); cabrerizopilar@gmail.com (P.C.-T.); s.gago.q@gmail.com (S.G.-Q.); dginel@yahoo.es (M.D.G.-F.); consuelojdf@gmail.com (C.J.); 2 Department of Legal Medicine, Psychiatry and Pathology, School of Medicine, Universidad Complutense de Madrid, 28040 Madrid, Spain; 3 Renal Physiopathology Laboratory, Department of Nephrology, Instituto de Investigación Sanitaria Gregorio Marañón, Hospital General Universitario Gregorio Marañón, 28040 Madrid, Spain; rengac@yahoo.es (M.Á.G.-N.); alberlaz@ucm.es (A.L.); 4 Department of Medicine, School of Medicine, Universidad Complutense de Madrid, 28040 Madrid, Spain; 5 Peritoneal and Retroperitoneal Unit, Department of Surgery, Hospital General Universitario Gregorio Marañón, 28040 Madrid, Spain; tumi100@icloud.com (W.V.-J.); lozanon57@hotmail.com (P.L.-L.); npalenciagarcia@gmail.com (N.P.-G.); 6 Department of Physiology, School of Medicine, Universidad Complutense de Madrid, 28040 Madrid, Spain; 7 Department of Surgery School of Medicine, Universidad Complutense de Madrid, 28040 Madrid, Spain

**Keywords:** cisplatin, imipenem/cilastatin, cilastatin, HIPEC, nephrotoxicity, nephroprotection

## Abstract

Cisplatin is one of the most widely used chemotherapeutic agents in oncology, although its nephrotoxicity limits application and dosage. We present the results of a clinical study on prophylaxis of cisplatin-induced nephrotoxicity in patients with peritoneal carcinomatosis undergoing cytoreduction and hyperthermic intraperitoneal intraoperative chemotherapy (HIPEC-cisplatin). Prophylaxis was with imipenem/cilastatin. Cilastatin is a selective inhibitor of renal dehydropeptidase I in the proximal renal tubule cells that can reduce the nephrotoxicity of cisplatin. Unfortunately, cilastatin is not currently marketed alone, and can only be administered in combination with imipenem. The study has a retrospective part that serves as a control (*n* = 99 patients receiving standard surgical prophylaxis) and a prospective part with imipenem/cilastatin prophylaxis corresponding to the study group (*n* = 85 patients). In both groups, we collected specific data on preoperative risk factors of renal damage, fluid management, hemodynamic control, and urine volume during surgery (including the hyperthermic chemotherapy perfusion), as well as data on hemodynamic and renal function during the first seven days after surgery. The main finding of the study is that cilastatin may exert a nephroprotective effect in patients with peritoneal carcinomatosis undergoing cytoreduction and hyperthermic intraperitoneal cisplatin perfusion. Creatinine values remained lower than in the control group (ANOVA test, *p* = 0.037). This translates into easier management of these patients in the postoperative period, with significantly shorter intensive care unit (ICU) and hospital stay.

## 1. Introduction

Peritoneal carcinomatosis is the growth of a tumor in the peritoneal surface, the inner surface of the abdomen. The tumor often arises from the epithelium of digestive or gynecologic organs, invades the wall of the organ, and sheds free tumor cells or tumor cell clusters into the peritoneal cavity. The small amount of physiologic peritoneal fluid in the cavity enables these cells to circulate freely in an attempt to find an adequate place to proliferate. Peritoneal implants develop in the right subdiaphragmatic space, greater omentum, bowel surfaces, mesentery, and pelvis and raise tumor masses throughout the peritoneal surface. Extensive tumor growth and poor prognosis of the disease have led to it being considered intractable and fatal [1].

In the late 1980s, Sugarbaker et al. [2] described the natural history of peritoneal carcinomatosis as a regional process that frequently remained in the abdominal cavity for a long period of time, even without distant metastasis. Based on this new concept, the authors developed a curative intent procedure for peritoneal malignant disease consisting of maximal cytoreductive surgery (CRS) to remove the whole macroscopic tumor followed by hyperthermic intraperitoneal chemotherapy (HIPEC) to remove microscopic residual disease [3,4]. HIPEC involves intraoperative perfusion of the abdominal cavity with a chemotherapy solution heated to 43 °C for 30–90 min. Residual tumor cells are exposed to the synergistic effect of chemotherapy and hyperthermia [5]. 

CRS + HIPEC has been adopted by many centers around the world. Many published studies show a change in the prognosis of peritoneal carcinomatosis based on this treatment, yielding very good results for peritoneal carcinomatosis from appendiceal mucinous tumors [6], ovarian carcinoma [7], peritoneal mesothelioma [8], and colorectal cancer [9,10].

Cisplatin (*cis*-diamminedichloroplatinum(II)) is commonly used in HIPEC for the treatment of epithelial ovarian carcinoma, fallopian tube carcinoma, primary peritoneal adenocarcinoma, malignant peritoneal mesothelioma, peritoneal sarcomatosis, and gastric cancer [11]. Its penetrative capacity and cytotoxicity are enhanced 3-fold by hyperthermia (41.5 °C) [11]. Nephrotoxicity is a major side effect of cisplatin that limits dosing and, therefore, the antitumor effect. Cisplatin accumulates in renal proximal tubular cells, resulting in inflammation, injury, and cell death and eventually acute kidney injury (AKI) [12]. The incidence of nephrotoxicity induced by HIPEC-cisplatin is variable, ranging between 4% and 30% [13,14], mainly because there is no standard definition of AKI, no standard classification (Risk Injury Failure Loss of kidney function and End-stage kidney disease (RIFLE), Acute Kidney Injury Network (AKIN), Common Terminology Criteria for Adverse Events (CTCAE)), and no standardization of the HIPEC procedure (e.g., cisplatin dose, concomitant drugs, length of perfusion). Furthermore, the development of AKI after CRS + HIPEC is affected by a series of preoperative factors (e.g., diabetes mellitus, arterial hypertension, angiotensin-converting enzyme inhibitors (ACE-I), angiotensin II receptor blockers (ARB), previous intravenous (IV) platinum cycles received, IV radiological contrasts) and intraoperative factors (e.g., volume and type of fluids, use of diuretics, use of vasoactive drugs).

Previous studies from our hospital in both in vitro and in vivo models have shown that cilastatin, a dipeptidyl analog that reversibly binds renal dehydropeptidase I (DHP-I) on the brush border of tubular cells, could be effective in preventing cisplatin-mediated nephrotoxicity by reducing apoptosis, oxidative stress, and inflammation [15,16,17,18], with no reduction in the effect of cisplatin on cancer cells [19]. Cilastatin, which was created to inhibit hydrolysis of the β-lactam ring of imipenem and prevent absorption of drugs into tubular cells [15], cannot yet be used as a nephroprotective agent in clinical practice because cilastatin is not available without imipenem (although a phase I safety trial was recently completed [20]). Clinical studies performed with imipenem/cilastatin (I/C) have shown the protective effect of cilastatin against cyclosporin-induced renal toxicity [21,22,23].

Few clinical studies assess prophylaxis for cisplatin-induced AKI in cancer patients. We performed a clinical study on advanced cancer patients undergoing HIPEC-cisplatin, with the main objective of assessing the effect of cilastatin (I/C) on the prevention of kidney damage.

## 2. Results and Discussion

We carried out a clinical study of patients with peritoneal carcinomatosis (mainly resulting from ovarian carcinoma), undergoing CRS + HIPEC-cisplatin with the aim of reducing the renal toxicity of cisplatin through the administration of I/C as antibiotic prophylaxis. The main finding of the study is that cilastatin may have a nephroprotective effect in CRS + HIPEC-cisplatin procedures. Clinical studies on prophylaxis of kidney damage with cisplatin are very scarce, thus highlighting the relevance of our research.

Peritoneal carcinomatosis occurs at an advanced stage of the neoplastic disease. Its traditionally poor short-term prognosis has improved significantly thanks to CRS + HIPEC. The surgical techniques are very invasive, patients are treated with IV chemotherapy (often with platinum drugs) before and after surgery, and it is therefore extremely important to preserve complete renal function for future management.

Perioperative AKI is a known problem in major abdominal surgery, and no therapeutic strategies to date have been shown to specifically protect the kidneys [24]. The use of HIPEC-cisplatin combined with major surgery may increase the problem. Dagel et al. [25] compared the incidence of AKI in patients with peritoneal or pleural carcinomatosis treated with IV cisplatin alone or HIPEC/hyperthermic intrathoracic chemotherapy (HITOC) with cisplatin or surgery alone and reported rates of 10.5%, 31.2%, and 11.7%, respectively. The authors concluded that the combination of anesthesia, major abdominal surgery, and cisplatin may have a synergistic effect on the development of AKI and that every effort should be made to decrease kidney damage [25].

### 2.1. Cisplatin-Induced Nephrotoxicity

Cisplatin is excreted almost exclusively by the kidneys. Cisplatin-induced kidney injury is mainly tubulointerstitial, because cisplatin concentrates at the proximal renal tubular cells at 5-fold higher concentrations than the peak serum concentrations. The risk of kidney injury increases with hypoalbuminemia, because free serum platinum levels increase [26]. It seems that hypomagnesaemia contributes to cisplatin-induced renal damage and that avoiding hypomagnesaemia may provide some nephroprotection [27]. Magnesium is critical to cellular homeostasis and enzymatic reactions and is a cofactor for ATP activity, mitochondrial respiration, and nucleic acid and protein synthesis. Hypomagnesaemia is a common manifestation of cisplatin-induced renal damage that is caused by increased loss of magnesium in urine (polyuria) and altered calcium metabolism.

Cisplatin has a high nephrotoxic potential that limits its dosing and anticancer effect. HIPEC-cisplatin takes advantage of one-shot administration immediately after complete cytoreduction when the residual tumor is minimal directly in the peritoneal cavity and boosted by hyperthermia. This approach seeks to increase the anticancer effect of cisplatin and to reduce nephrotoxicity based on local administration and the plasma–peritoneal barrier [5]. The antitumor efficacy of cisplatin administered during HIPEC has been shown by detecting DNA-adducts in tumor samples removed during the procedure [28]. HIPEC-cisplatin at 100 mg/m^2^ achieves a perfusate/plasma area under the curve of up to 19.5 [28]. Therefore, the peritoneal concentration is almost 20 times higher than in plasma, although as the concentration here is not zero, nephrotoxicity is not totally avoided.

Three factors highlight the importance of AKI during HIPEC, as follows: (1) the more frequent use of specific AKI scales such as RIFLE has revealed that it can affect 30% of patients undergoing HIPEC [14]; (2) AKI induced by HIPEC increases postoperative complexity, intensive care unit (ICU) and hospital stay, and mortality, although it can also cause permanent reduction in the kidney function that will hinder subsequent treatment with cisplatin and other types of chemotherapy [26]; (3) The current trend in the management of fluid therapy for hemodynamic objectives in anesthesia moves us away from the classic objective of hydration during HIPEC and may further compromise renal function [29]. Hydration is a generally indicated measure that significantly decreases the half-life of cisplatin, its urinary concentration, and transit time in the proximal tubule. It seems that hydration is essential even with low doses of cisplatin [11,12,13]. Cisplatin-induced nephrotoxicity can also be reduced by administration of thiosulfate, which is a chelator of cisplatin through its thiol group (-SH) in the bloodstream, forming inactive compounds that are not toxic to the kidneys, but may simultaneously decrease the antitumor efficacy of cisplatin [30]. In the clinical trial by van Driel et al. [7], systematic nephroprotection with sodium thiosulfate was used in the HIPEC group via IV infusion during HIPEC and up to three hours later. The authors did not report nephrotoxicity in the HIPEC group. Along the same lines, the use of amifostine as a renal protector has also been proposed [31]. Amifostine is a thiophosphate that is metabolized by alkaline phosphatase to a thiol product capable of binding metabolites of platinum and free radicals, which may accelerate DNA repair in normal cells. The main side effect of amifostine is severe hypotension [32], which is a major handicap in a complex surgical procedure such as CRS + HIPEC. Furthermore, as with thiosulfate, tumor protection cannot be excluded.

Older studies have shown that the use of I/C as a prophylactic antibiotic in heart, kidney, and bone marrow recipients was able to decrease cyclosporine-induced kidney damage [21,22,23,33]. Likewise, experimental studies in rats have shown that cilastatin may protect from cisplatin-induced kidney injury by reducing the expansion phase of AKI without modifying its antitumor effect [15,19].

Some studies have identified specific AKI risk factors in CRS + HIPEC (age, obesity) and HIPEC-cisplatin such as previous impairment of renal function, high number of cycles of IV chemotherapy, short interval between IV chemotherapy and surgery, need for perioperative transfusion, low intraoperative urine output, angiotensin II receptor antagonist use, and hypertension [26,34,35,36].

### 2.2. Baseline Characteristics of the Study

**Figure 1:**
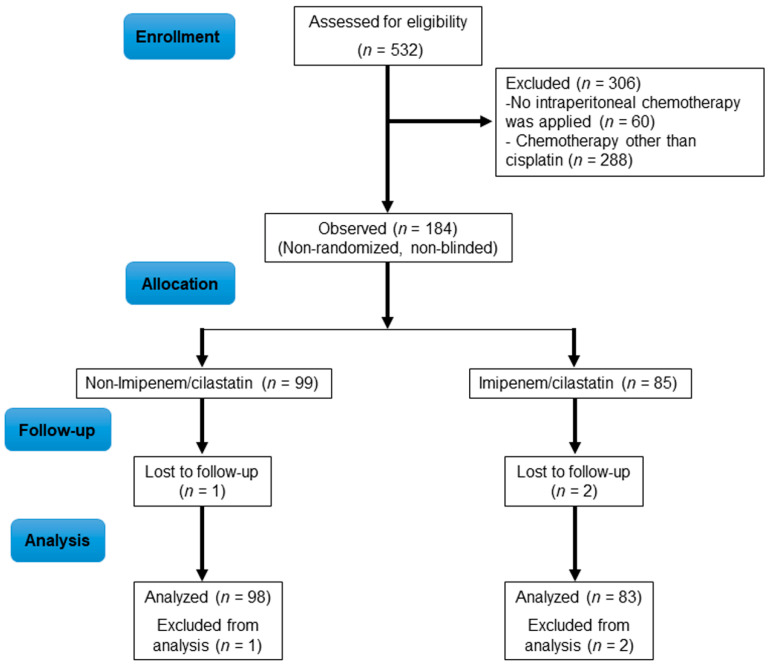
Flowchart of patient enrolment and follow-up.

The malignant peritoneal disease program at our institution treated 532 patients with CRS + HIPEC from January 2011 to September 2020. Of these, 184 underwent CRS + HIPEC-cisplatin. The retrospective part of the study ran from January 2011 to December 2015 and included 99 patients treated with CRS + HIPEC-cisplatin with regular antibiotic prophylaxis (non-I/C group) (Figure 1). The prospective part of the study ran from January 2016 to September 2020 and included 85 patients who underwent CRS + HIPEC-cisplatin with imipenem/cilastatin as antibiotic prophylaxis (I/C group) (Figure 1). Three patients were excluded, two in the I/C group and one in the non-I/C group, because of deviation from the anesthetic protocol resulting from increased diuresis during the HIPEC phase (Figure 1).

Demographic, oncological, surgical, and anesthetic characteristics of the population are shown in Table 1**.** The two groups were fairly similar: almost the whole study sample was made up of women, since the most common tumor treated with CRS + HIPEC-cisplatin is epithelial ovarian carcinoma. Body mass index (BMI) and body surface area (BSA) were homogeneous. More importantly, the extent of peritoneal disease was the same, as were previous treatments with platins and comorbidity related to nephrotoxicity (chronic kidney disease, diabetes mellitus, hypertension, previous treatment with ACE-I, ARB, non-steroidal anti-inflammatory drugs (NSAIDs) and IVP contrast media during the days before surgery) was similar between both groups.

Patients in the I/C group were significantly older, and a lower proportion were American Society of Anesthesiologists (ASA) III; this finding was related to changes in our patient selection protocol. Interestingly, our group participated in a study showing good results in patients aged more than 75 years with good performance status [37]. AKI was more likely to be associated with poorer performance status in older patients. However, the results we report here differ, indicating CRS + HIPEC-cisplatin is useful in elderly patients.

### 2.3. Surgical and Anesthetic Data

Anesthesia and surgery were shorter in the I/C group, mainly due to the reduction in the duration of HIPEC from 90 min to 60 min and to changes in the surgical reconstruction phase (anastomosis), which began to be performed before HIPEC, thus shortening time (Table 1).

Perioperative fluid infusion was significantly reduced in the I/C group based on the reduction in crystalloids and the shorter duration of anesthesia and surgery, although total perioperative urine output and HIPEC urine output remained unchanged (Table 1). Following current protocols for major surgery, restrictive fluid administration can potentially help to mitigate morbidity in patients undergoing CRS + HIPEC [38]. However, this practice goes against classic active hydration to increase the volume of diuresis and thus reduce the half-life of cisplatin, its urinary concentration, and transit time in the proximal tubule [39]. In any case, it has been accepted as a standard for reducing associated nephrotoxic effects.

In our protocol, the goal of diuresis during the HIPEC period was around 150 mL/15 min urine output, based on aggressive administration of fluids and diuretics (Table 1). The fluid management strategy in the pre- and post-HIPEC phase followed the principles of goal-directed therapy (GDT). Compared with conventional treatment, GDT has proven effective for reducing postoperative complications and even mortality in “high-risk” patients undergoing major abdominal surgery and in patients undergoing CRS + HIPEC [29,40]. Forced diuresis using mannitol or furosemide with adequate hydration has been shown to be beneficial when high doses of cisplatin are used [41]. These strategies have broad implications for clinical practice and represent the best practice principles for the prevention of cisplatin-induced nephrotoxicity. In our study, the anesthetic HIPEC team applied this mechanism of nephroprotection in all cases. Consequently, there were no differences between the two groups in the total volume of urine output throughout surgery and during the HIPEC period (Table 1).

In the context of the complexity of CRS + HIPEC, it is important to evaluate the hemodynamic support provided by vasoactive agents. We found no differences in the administration of vasopressors between the two groups, suggesting that the impact and aggressiveness of the procedure and patient response were similar in both groups (Table 1).

### 2.4. HIPEC Procedure

HIPEC was always performed using the open coliseum technique, with 2 L/m^2^ dialysate perfusate and 100 mg/m^2^ cisplatin. The temperature goal was 42 °C in the abdominal cavity throughout the intraperitoneal perfusion. HIPEC lasted 60–90 min.

There were more cases of HIPEC-cisplatin + doxorubicin in the non-I/C group than in the I/C group, owing to the change of protocol in 2015 (Table 1). Doxorubicin is metabolized in the liver and excreted with bile and feces. Therefore, it does not cause kidney damage or interfere with elimination of cisplatin.

There were no differences in the cisplatin dose administered during HIPEC in either group (total dose and per m^2^ of body surface area, Table 1).

### 2.5. Morbidity and Mortality

The use of imipenem in surgical antibiotic prophylaxis is clearly not adequate. It is a reserve drug for bacterial infections resistant to common antibiotics, and its indication is based on local antibiotic guidelines. The justification for using it in our study was its association with cilastatin, which could have nephroprotective benefits for the patient. One possible drawback is that of bacterial resistance. For this reason, administration was strictly reduced to three doses, which were administered during the surgical procedure. This regimen does not place significant pressure on the bacterial population in terms of resistance. In addition, the duration of administration is too short for carbapenemases to develop or for bacterial permeability to antibiotics to decrease [42]. In our study, we did not observe differences between groups in surgical site infections (SSI) (superficial or deep incisional SSI and organ or space SSI) in relation to antibiotic prophylaxis.

There were significantly more major complications (grade 3 and 4 (Clavien–Dindo classification)) in the non-I/C group than in the I/C group, although mortality was similar (Table 1). The study clearly shows a significant reduction in ICU stay (*p* = 0.02) and hospital stay (*p* = 0.005) in the I/C group, possibly as a consequence of the reduction in AKI in these patients (Table 1). Several studies have shown that AKI increases morbidity and mortality [43,44] and ICU and hospital stay [14]. In fact, the development of AKI can even increase mortality in the short term [45] and the risk of developing chronic kidney disease [46].

### 2.6. Renal Function and Protection with Cilastatin

I/C is a powerful antibiotic that was first marketed in the 1980s. Cilastatin was created specifically to inhibit the DHP-I enzyme from the brush border of proximal tubule cells, which are located in membrane domains known as cholesterol lipid rafts and responsible for hydrolyzing the β-lactam ring of imipenem, thus inactivating it [15]. Therefore, cilastatin prevents absorption of imipenem by increasing urinary excretion and reducing the concentration inside the tubular cells. Previous studies at our hospital and elsewhere showed that cilastatin is effective for reducing the nephrotoxicity of various drugs such as antibiotics [47,48], immunosuppressants [49], analgesics [50], and chemotherapeutics without modifying their therapeutic efficacy in target cells. Cilastatin has been shown to protect proximal tubular cells from apoptotic, oxidative, and inflammatory toxic damage induced by cisplatin both in vitro and in vivo by reducing or preventing cisplatin-induced AKI and its worsening [15,16,17,18,19]. Our study demonstrates this reduction in AKI.

One of the amplification mechanisms of cisplatin-induced AKI is based on the formation of a Fas/Fas ligand (FasL) complex on the surface of the renal cells adjacent to the initial injured cell, and specifically in cholesterol rafts, that triggers the extrinsic pathway of apoptosis [51], thus perpetuating kidney damage. The protective effect of cilastatin is directly related to the interruption of lipid raft cycling, which inhibits internalization of Fas–FasL bound to cell membrane cholesterol lipid rafts [15,16,19]. This effect decreases the levels of both Fas and FasL, thus preventing the extrinsic pathway of apoptosis and reducing activation of caspase 8, 3, and 9, mitochondrial depolarization, extrusion of cytochrome c into the cytosol, endonuclease activity, and oxidative and proinflammatory NF-κB activation, thereby protecting tubular cells [15,16,19]. Other treatments, such as medicinal herbs (e.g., *Hydrangea paniculata*), have shown very similar effects in protecting against the kidney damage resulting from inhibition of the elevation of the Fas/FasL system by cisplatin [52].

Other cilastatin-mediated protective mechanisms play an important role in renal protection against cisplatin. These include direct blockade of megalin (also in cholesterol rafts), as reported by Hori et al. [53].

To the best of our knowledge, this is the first clinical study to use cilastatin as a protector against cisplatin-induced AKI. Our clinical study revealed a nephroprotective effect of cilastatin against cisplatin in cancer patients undergoing CRS + HIPEC. In cancer, cilastatin has the appeal of not interfering with the cytotoxic effect of cisplatin and not generating collateral toxicity. As mentioned above, van Driel et al. [7] treated stage III ovarian carcinoma with and without HIPEC-cisplatin (100 mg/m^2^) using sodium thiosulfate as a nephroprotective agent. The authors did not report nephrotoxicity in the HIPEC group, although this strategy may have decreased the anti-tumor efficacy of cisplatin.

**Figure 2:**
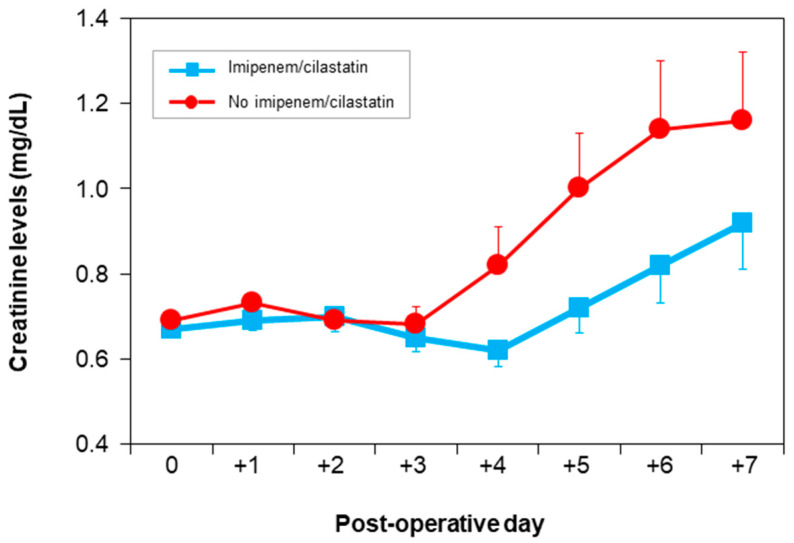
Change in serum creatinine levels from baseline up to day 7 in both groups.

Postoperative renal function was evaluated by analyzing the values of postoperative creatinine levels in both groups. Postoperative serum creatinine levels differed significantly between both groups (ANOVA test; *p* = 0.037). Figure 2 shows the nephroprotective effect of I/C, which maintains creatinine values closer to the normal range.

Detailed day-to-day analysis revealed significant differences in creatinine levels on day 4 (0.62 ± 0.33 vs. 0.82 ± 0.78 mg/dL; *p* = 0.04) and differences that were at the limit of statistical significance on day 5 (0.72 ± 0.5 vs. 1 ± 1 mg/dL; *p* = 0.06) and on day 6 (0.82 ± 0.67 vs. 1.14 ± 1.2; *p* = 0.09) (Figure 2 and Table 2).

These results are consistent with those of previous studies, showing that AKI is induced by cisplatin on day 4–5 of administration, with diuresis generally preserved [26].

**Figure 3:**
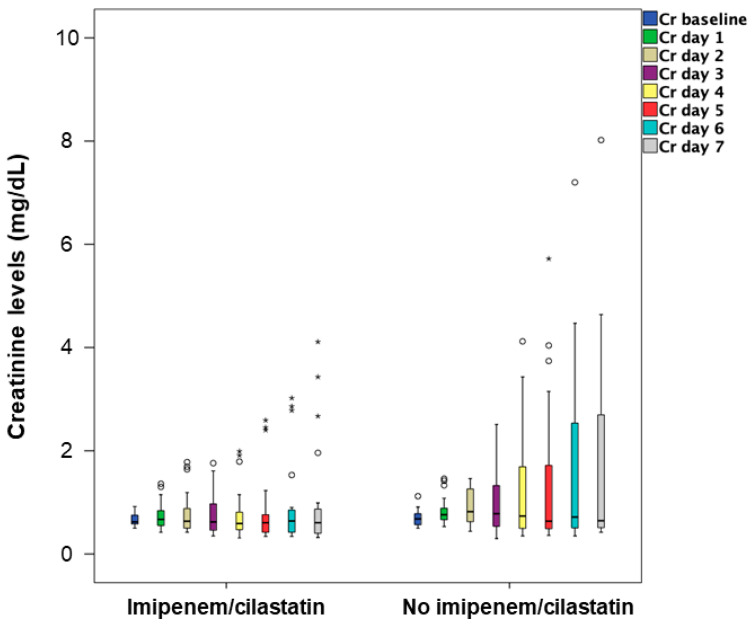
Boxplot of serum creatinine from baseline and up to day 7 in both groups. Boxes show the median and the interquartile range. Whiskers show upper and lower adjacent values.

A plot of these values adequately reflects the pathophysiology of AKI after only one dose of 100 mg/m^2^ cisplatin in both groups (Figure 3).

**Figure 4:**
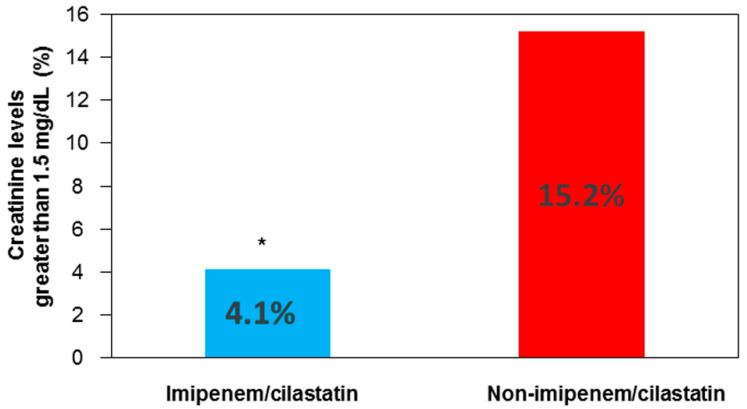
Percentage of patients with creatinine levels above 1.5 mg/dL on day 4 of the postoperative period in both groups. * *p* = 0.024 vs. the non-imipenem/cilastatin group.

The analysis of the percentage of patients presenting creatinine levels above 1.5 mg/dL on day 4 revealed significant differences (Figure 4).

However, in our study, the incidence of some degree of AKI according to the RIFLE classification was 25.5% in non-I/C group and 22.8% in the I/C group (non-significant) (Table 3). This may be due to the limitations of the RIFLE classification, including the fact that it does not take into account the etiology of AKI. Furthermore, this classification does not provide information regarding the origin of the injury [54].

### 2.7. Limitations of the Study

Our study is subject to a series of limitations. It took 10 years (five for the retrospective part and five for the prospective part), and the results could have been affected by changes in anesthetic and surgical protocols during this time. Furthermore, the absence of a commercial formulation of cilastatin obliged us to use doses of I/C set out in the antibiotic prophylaxis policy of our institution. Our previous studies on nephroprotection with cilastatin and the successful completion of a phase I clinical trial make the marketing of the drug a real possibility, on the condition that its clinical efficacy can be demonstrated [20]. Our randomized clinical trial on the nephroprotective role of cilastatin (I/C) against cisplatin administered by HIPEC resolves doubts about the future protective effect of cilastatin. We believe that cilastatin can become a “renal omeprazole” in clinical practice.

## 3. Materials and Methods

**Figure 5:**
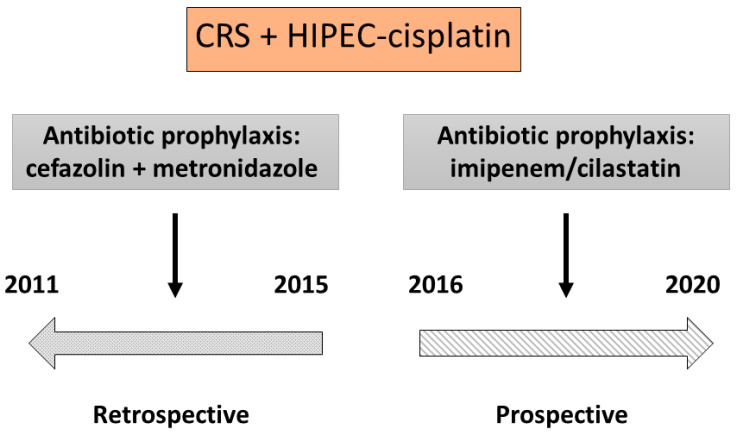
Time sequence of the study protocol. CRS, cytoreductive surgery; HIPEC, hyperthermic intraperitoneal chemotherapy.

To study the role of cilastatin as a nephroprotective agent in patients receiving CRS + HIPEC-cisplatin, we designed a study with two parts: a retrospective part, running from 2011 to 2015 and including patients receiving CRS + HIPEC-cisplatin with usual prophylactic antibiotics (cefazolin repeated every four hours + metronidazole every six hours during surgery), i.e., the “non-I/C group”; and a prospective part, running from 2016 to 2020 and including patients receiving CRS + HIPEC-cisplatin with I/C as antibiotic prophylaxis (three doses of 500/500 mg I/C, the first during induction of anesthesia, the second before the beginning of HIPEC-cisplatin, and the third in the ICU, 6 h after the second dose), i.e., the “I/C group” (Figure 5). The Microbiology Department was consulted about the use of I/C as a prophylactic antibiotic, and we were authorized to use a maximum of three doses of I/C from induction of anesthesia up to 24 h. The study was approved by the Ethics Committee of Gregorio Marañón Hospital with registration code NEFROHIPEC 016 and was conducted in accordance with the Declaration of Helsinki.

Patients with peritoneal carcinomatosis were evaluated by the Multidisciplinary Committee on Peritoneal Malignant Disease at our institution. The study population comprised patients for CRS + HIPEC-cisplatin during the indicated periods. Patients mainly had epithelial ovarian carcinoma, although some had peritoneal mesothelioma and gastric cancer. 

### 3.1. Anesthesia

The anesthesia team followed the institution’s protocols during the study period, including administration of general and epidural anesthesia. General anesthesia was continued with propofol or sevoflurane, fentanyl or remifentanil, and rocuronium throughout the procedure. In addition to standard anesthetic monitoring, patients underwent advanced hemodynamic monitoring with invasive assessment of arterial blood pressure and cardiac output using the Flotrac-Vigileo monitor or VolumeView/EV1000™ system (Edwards Lifesciences, Irvine, CA, USA). Depth of anesthesia was monitored using the bispectral index. Esophageal and vesical temperature was monitored throughout the procedure. Fluids were administered following the protocols of GDT by the regular anesthesia team in charge of HIPEC. Immediately before HIPEC, intravenous fluid administration was increased to maintain a urine output of 150 mL/15 min, and furosemide was administered when necessary. Hemoglobin in the amount of 8–9 g/dL was considered the transfusion threshold. Other agents, such as albumin, magnesium, potassium, and calcium, were administered when required.

### 3.2. CRS + HIPEC

The surgical procedure consisted of four phases: (1) evaluation of the extension of peritoneal disease based on the peritoneal cancer index (PCI) of Sugarbaker [55]; (2) CRS and assessment of completeness of cytoreduction based on the completeness of cytoreduction score (CC score) described by Sugarbaker [56]; (3) HIPEC; and (4) digestive reconstruction (anastomosis). A xyphopubic laparotomy was performed in phase 1, and peritoneal disease was staged using the PCI of Sugarbaker. Implants were graded according to size in each of the 13 areas of the abdomen as follows: <5 mm, 1 point; 6 mm–5 cm, 2 points; >5 cm or confluent lesions, 3 points (maximum score, 39). In phase 2, CRS was performed by parietal peritonectomy of the affected areas [4], resection of affected viscera, and electroevaporation of tumor implants. After surgery, the degree of cytoreduction achieved was evaluated according to the CC score (CC0 = no macroscopic residual disease; CC1 = macroscopic residual tumor up to <2.5 mm; CC2, >2.5 mm and ≤2.5 cm; CC3 ≥ 2.5 cm). In phase 3, HIPEC was administered following the Coliseum technique [57]. The autostatic retractor is lifted 20–25 cm from the patient and the skin edge of the incision is suspended by a suture. The suture also fixes a plastic sheet to the open surgical wound to protect the surgical team from splashes. This sheet is opened in its center to establish the perfusion circuit and stir the perfusate inside the abdomen so that it is fully distributed. A perfusion circuit is established with four percutaneous outflow drains that join into an outflow line and one inflow catheter that passes through a heat exchanger, and the circuit is driven by two roller pumps. We placed two temperature sensors in the abdomen, one in the right subphrenic space and the other in the pelvis. The heat exchanger raises the perfusate temperature to 44–46 °C to maintain the intraperitoneal fluid at 42 °C. The perfusate is a peritoneal dialysis solution of 1.35% glucose with a volume of 2 L/m^2^ of body surface. The BSA was calculated according to Mosteller’s formula in Equation (1):

(1) $$BSA\left(m^{2}\right)=\sqrt{\frac{Ht\;\left(cm\right)\times Wt\;\left(kg\right)}{3600}}$$

Cisplatin was requested from the Pharmacy Service at a dose of 100 mg/m^2^ and was sent to the operating room in 250 cc of saline. The dose was reduced by 25% in patients older than 65 years, patients who had previously received more than four cycles of platinum, creatinine clearance <87 mL/min (lower limit of norm for our laboratory), surgery longer than 6 h, or aggressive surgery/visceral resections. Cisplatin was added to the perfusate when the intraabdominal temperature reached ≥42 °C. The perfusion lasted 60–90 min. Efforts were made during HIPEC to maintain diuresis at 150 cc/15 min to avoid renal toxicity of cisplatin. In phase 4, after HIPEC, the perfusion circuit was removed, drains were left in place, and the necessary anastomoses were performed to complete the intervention. After the procedure, the patient was transferred to the postoperative care unit until recovery from anesthesia and then moved to the ward.

### 3.3. Changes in the HIPEC Protocol

In 2015, we changed our protocol by performing phase 4 reconstruction before phase 3 HIPEC based on the observation that after HIPEC, the bowel wall was edematous. We therefore thought it would be better to perform the anastomosis before this happened. Similarly, since 2015, we progressively reduced the length of HIPEC from 90 to 60 min based on the results of pharmacokinetics studies [58]. Before 2015, it was common to use HIPEC-cisplatin + doxorubicin for the treatment of ovarian carcinoma; since then, we have removed doxorubicin from intraperitoneal perfusion because it created compatibility problems with subsequent chemotherapy regimens.

### 3.4. Data Collection

The data collected included age, sex, weight, height, BMI, BSA, ASA class, comorbidity associated with nephrotoxicity (chronic kidney diseases, diabetes mellitus, hypertension, previous treatment with ACE-I, ARB, NSAIDs, and intravenous contrast media received in the days before the surgery), primary tumor, extent of peritoneal damage, and intraoperative data: PCI, duration of the procedure, duration of anesthesia, dose of cisplatin, previous cisplatin chemotherapy, blood transfusion and fresh frozen plasma requirements, volume of crystalloids and colloids infused perioperatively, use of vasopressor and diuretics agents, and serum creatinine levels from before surgery to the seventh day after surgery.

### 3.5. Statistical Analysis

The statistical analysis was performed using IBM SPSS Statistics for Windows, v. 22.0 (IBM Corp., Armonk, NY, USA). Continuous data were first analyzed for normality using the Kolmogorov–Smirnov test. Quantitative variables are shown as the means ± standard deviation (SD) and qualitative variables are shown as numbers (percentage). A t-test was used for quantitative variables and a chi-squared test or Fisher’s exact test was used for categorical variables. Repeated measures ANOVA was used to compare postoperative creatinine levels between the groups. The differences were considered statistically significant if the *p*-value was less than 0.05.

### 3.6. Sample Size

Previous studies showed that HIPEC-cisplatin is associated with an incidence of AKI ranging from 4% to 30%. Accepting an alpha risk of 0.05 and a beta risk of 0.2 in a two-sided test, a minimum of 78 subjects are necessary in the first group and 78 in the second to establish a statistically significant proportional difference, which was expected to be 0.25 in the non-I/C group and 0.08 in the I/C group.

## 4. Conclusions

We performed a clinical study in patients with peritoneal carcinomatosis resulting mainly from epithelial ovarian carcinoma who were undergoing CRS + HIPEC-cisplatin. Our objective was to reduce renal toxicity of cisplatin through the administration of I/C as antibiotic prophylaxis. The main finding of the study was that cilastatin seems to have a nephroprotective effect in CRS + HIPEC-cisplatin procedures. Clinical studies on cisplatin for prophylaxis of kidney injury are very scarce, thus highlighting the relevance of our research.

Peritoneal carcinomatosis is an advanced stage of neoplastic disease with a compromised prognosis in the short term. Treatment has improved significantly with CRS + HIPEC, with cisplatin being one of the most widely used chemotherapeutic agents in HIPEC. However, the main toxicity of cisplatin is renal, thus limiting the dose administered and the duration of treatment. CRS + HIPEC is a maximally invasive procedure that is performed in patients heavily treated with chemotherapy, mainly platins, before and after the procedure. Therefore, it is very important to preserve complete renal function for future management. In the search for an ideal nephroprotective agent, we conceived a drug that does not interfere with the cytotoxic action of the chemotherapeutic and acts selectively in the kidney without producing side effects. Cilastatin acts in the proximal renal tubule, where it binds reversibly to DHP-I on the ciliated border of cells and can reduce the inflammation, oxidative stress, and apoptosis caused by cisplatin without reducing its antitumor capacity. Therefore, cilastatin may represent a new therapeutic strategy for preservation of renal function in cisplatin-treated cancer patients.

## 5. Patents

The following patents are in part related to the work reported in this manuscript:

“Use of cilastatin to reduce nephrotoxicity of various compounds,” patent numbers EP 2143429 B1; US 9,216,185 B2; US 9,522,128 B2; and US-9757349-B2. Patents are assigned to Fundación para la Investigación Biomédica del Hospital Gregorio Marañón (FIBHGM) and licensed by FIBHGM to Telara Pharma S.L.

## Figures and Tables

**Table 1:** Baseline characteristics.

Patient Characteristics	Non-I/C Group (*n* = 98)	I/C Group (*n* = 83)	*p*-Value
Age (years)	53.22 ± 10.94	56.79 ± 11.42	0.034
Patients > 65 years	12 (12.4)	21 (25.3)	0.025
ASA, *n* (%)			
I	1 (1)	2 (2.4)	0.06
II	69 (70.4)	69 (83.1)	
III	28 (28.6)	12 (14.5)	
Sex, *n* (%)			
Female	91 (92.9)	80 (94)	0.76
Male	7 (7.1)	5 (6)	
Comorbidity associated with nephrotoxicity, *n* (%)	23 (24)	23 (27.7)	0.56
BMI (kg/m^2^)	24.5 ± 6.29	25.4 ± 5.3	0.33
BSA (m^2^)	1.65 ± 0.17	1.63 ± 0.21	0.47
Previous chemotherapy with cisplatin	71 (72.4)	67 (80.7)	0.12
Number of cycles of previous cisplatin chemotherapy	4.35 ± 3.78	4.21 ± 2.5	0.76
Tumor:	-	-	0.87
Ovarian	82	69	-
Colon adenocarcinoma	1	1	-
Appendix	2	1	-
Gastric tumor	1	3	-
Mesothelioma	10	8	-
Other	2	1	-
PCI	15.36 ± 10.23	15.25 ± 9.80	0.94
Duration of anesthesia (min)	682.7 ± 127.77	614.28 ± 105.90	0.0001
Duration of surgery (min)	566.29 ± 126.13	497.39 ± 101.87	0.0001
Duration of HIPEC (min)	68.50 ± 13.84	60.72 ± 6.5	0.0001
Perioperative urine output (mL)	1223.33 ± 387.08	1464.28 ± 592.36	0.18
Urine output during HIPEC (mL)	891.39 ± 375.51	845.48 ± 354.55	0.40
Urine output during HIPEC > 150 mL/15 min, *n* (%)	70 (76.1)	66 (80.5)	0.60
Intraoperative administration of diuretic (%)	53 (71.6)	66 (82.5)	0.14
Perioperative fluid balance (mL)	6897.22 ± 2849.94	5127.81 ± 1493.38	0.0001
Crystalloids (mL)	5781.25 ± 2516.55	3905.06 ± 1220.63	0.0001
Colloids, *n* (%)	66 (66)	46 (54)	0.31
Vasopressor, *n* (%)	17 (17.1)	25 (29)	0.11
Dose of cisplatin (mg)	142.23 ± 34.35	142.09 ± 32.28	0.97
Dose of cisplatin (mg/BSA)	85.27 ± 18.86	87.21 ± 18.5	0.50
Cisplatin + doxorubicin (%)	53 (53.5)	8 (9.4)	0.0001
Stay in intensive care > 3 days, *n* (%)	27 (27.6)	12 (14.1)	0.02
Length of hospital stay	24.11 ± 30	13.52 ± 10.9	0.005
90-day mortality, *n* (%)	3 (3)	0	0.15
Major complications, *n* (%)	18 (18.4)	8 (9.6)	0.07

Data are presented as the means ± standard deviation (SD) or absolute numbers (%). I/C, imipenem/cilastatin; BMI, body mass index; BSA, body surface area; PCI, peritoneal cancer index; ASA, American Society of Anesthesiologists; HIPEC, hyperthermic intraperitoneal chemotherapy.

**Table 2:** Serum creatinine levels (mg/dL) from baseline up to day 7.

Time	Non-I/C Group (*n* = 98)	I/C Group (*n* = 83)	*p*-Value
Baseline	0.69 ± 0.13	0.67 ± 0.13	0.57
Day 1	0.73 ± 0.23	0.769 ± 0.22	0.31
Day 2	0.69 ± 0.25	0.70 ± 0.32	0.89
Day 3	0.68 ± 0.40	0.65 ± 0.30	0.58
Day 4	0.82 ± 0.78	0.62 ± 0.33	0.04 *
Day 5	1.00 ± 1.02	0.72 ± 0.51	0.06 **
Day 6	1.14 ± 1.25	0.82 ± 0.67	0.09 **
Day 7	1.16 ± 1.28	0.92 ± 0.89	0.2

Data are presented as the means ± SD. * Statistically significant; ** at the limit of statistical significance. I/C, imipenem/cilastatin.

**Table 3:** RIFLE classification.

Category	Non-I/C Group (*n* = 98)	I/C Group (*n* = 83)	*p*-Value
No renal failure	73	64	0.83
Risk	8	7	
Injury	10	6	
Failure	3	4	
Loss	4	2	
ESRD	-	-	

RIFLE, Risk Injury Failure Loss of kidney function and End-stage kidney disease; ERSD, end-stage renal disease; I/C, imipenem/cilastatin.

## Data Availability

The data that support the findings presented in this manuscript are available from the corresponding author upon reasonable request.

## Abbreviations

ACE-I	Angiotensin-converting enzyme inhibitors
AKI	Acute kidney injury
AKIN	Acute Kidney Injury Network
ARB	Angiotensin II receptor blockers
ASA	American Society of Anesthesiologists
BMI	Body mass index
BSA	Body surface area
CC score	Completeness of cytoreduction score
CRS	Cytoreductive surgery
CTCAE	Common Terminology Criteria for Adverse Events
DHP-I	Dehydropeptidase I
HIPEC	Hyperthermic intraperitoneal chemotherapy
HITOC	Hyperthermic intrathoracic chemotherapy
I/C	Imipenem/cilastatin
ICU	Intensive care unit
PCI	Peritoneal cancer index
RIFLE	Risk, Injury, Failure, Loss of kidney function, and End-stage kidney disease

## References

[1] Sadeghi B., Arvieux C., Glehen O., Beaujard A.C., Rivoire M., Baulieux J., Fontaumard E., Brachet A., Caillot J.L., Faure J.L. (2000). Peritoneal carcinomatosis from non-gynecologic malignancies: Results of the EVOCAPE 1 multicentric prospective study. Cancer.

[2] Sugarbaker P.H., Cunliffe W.J., Belliveau J., de Bruijn E.A., Graves T., Mullins R.E., Schlag P. (1989). Rationale for integrating early postoperative intraperitoneal chemotherapy into the surgical treatment of gastrointestinal cancer. Semin. Oncol..

[3] Jacquet P., Sugarbaker P.H., Sugarbaker P.H. (1996). Clinical research methodologies in diagnosis and staging of patients with peritoneal carcinomatosis. Peritoneal Carcinomatosis: Principles of Management.

[4] Sugarbaker P.H. (1995). Peritonectomy procedures. Ann. Surg..

[5] González-Moreno S., González-Bayón L.A., Ortega-Pérez G. (2010). Hyperthermic intraperitoneal chemotherapy: Rationale and technique. World J. Gastrointest. Oncol..

[6] Yan T.D., Bijelic L., Sugarbaker P.H. (2007). Critical analysis of treatment failure after complete cytoreductive surgery and perioperative intraperitoneal chemotherapy for peritoneal dissemination from appendiceal mucinous neoplasms. Ann. Surg. Oncol..

[7] Van Driel W.J., Koole S.N., Sikorska K., Schagen van Leeuwen J.H., Schreuder H.W.R., Hermans H.M.R., de Hingh I.H.J.T., van der Velden J., Arts H.J., Massuger L.F.A.G. (2018). Hyperthermic Intraperitoneal Chemotherapy in Ovarian Cancer. N. Engl. J. Med..

[8] Yan T.D., Deraco M., Baratti D., Kusamura S., Elias D., Glehen O., Gilly F.N., Levine E.A., Shen P., Mohamed F. (2009). Cytoreductive surgery and hyperthermic intraperitoneal chemotherapy for malignant peritoneal mesothelioma: Multi-institutional experience. J. Clin. Oncol..

[9] Elias D., Gilly F., Boutitie F., Quenet F., Bereder J.M., Mansvelt B., Lorimier G., Dubè P., Glehen O. (2010). Peritoneal colorectal carcinomatosis treated with surgery and perioperative intraperitoneal chemotherapy: Retrospective analysis of 523 patients from a multicentric French study. J. Clin. Oncol..

[10] Nadler A., McCart J.A., Govindarajan A. (2015). Peritoneal Carcinomatosis from Colon Cancer: A Systematic Review of the Data for Cytoreduction and Intraperitoneal Chemotherapy. Clin. Colon Rectal Surg..

[11] Takemoto M., Kuroda M., Urano M., Nishimura Y., Kawasaki S., Kato H., Okumura Y., Akaki S., Kanazawa S., Asaumi J. (2003). The effect of various chemotherapeutic agents given with mild hyperthermia on different types of tumours. Int. J. Hyperth..

[12] Oh G.S., Kim H.J., Shen A., Lee S.B., Khadka D., Pandit A., So H.S. (2014). Cisplatin-induced Kidney Dysfunction and Perspectives on Improving Treatment Strategies. Electrolyte Blood Press..

[13] Canda A.E., Sokmen S., Terzi C., Arslan C., Oztop I., Karabulut B., Ozzeybek D., Sarioglu S., Fuzun M. (2013). Complications and toxicities after cytoreductive surgery and hyperthermic intraperitoneal chemotherapy. Ann. Surg. Oncol..

[14] Arjona-Sánchez A., Cadenas-Febres A., Cabrera-Bermon J., Muñoz-Casares F.C., Casado-Adam A., Sánchez-Hidalgo J.M., López-Andreu M., Briceño-Delgado J., Rufián-Peña S. (2016). Assessment of RIFLE and AKIN criteria to define acute renal dysfunction for HIPEC procedures for ovarian and non ovarian peritoneal malignances. Eur. J. Surg. Oncol..

[15] Camaño S., Lazaro A., Moreno-Gordaliza E., Torres A.M., de Lucas C., Humanes B., Lazaro J.A., Gomez-Gomez M.M., Bosca L., Tejedor A. (2010). Cilastatin attenuates cisplatin-induced proximal tubular cell damage. J. Pharmacol. Exp. Ther..

[16] Humanes B., Camaño S., Lara J.M., Sabbisetti V., González-Nicolás M.A., Bonventre J.V., Tejedor A., Lázaro A. (2017). Cisplatin-induced renal inflammation is ameliorated by cilastatin nephroprotection. Nephrol. Dial. Transplant..

[17] Moreno-Gordaliza E., Esteban-Fernández D., Lázaro A., Aboulmagd S., Humanes B., Tejedor A., Linscheid M.W., Gómez-Gómez M.M. (2018). Lipid imaging for visualizating cilastatin amelioration of cisplatin-induced nephrotoxicity. Lipid Res..

[18] Moreno-Gordaliza E., Giesen C., Lázaro A., Esteban-Fernández D., Humanes B., Cañas B., Panne U., Tejedor A., Jakubowski N., Gómez-Gómez M.M. (2011). Elemental bioimaging in kidney by LA-ICP-MS as a tool to study nephrotoxicity and renal protective strategies in cisplatin therapies. Anal. Chem..

[19] Humanes B., Lazaro A., Camaño S., Moreno-Gordaliz E., Lazaro J.A., Blanco-Codesido M., Lara J.M., Ortiz A., Gomez-Gomez M.M., Martín-Vasallo P. (2012). Cilastatin protects against cisplatin-induced nephrotoxicity without compromising its anticancer efficiency in rats. Kidney Int..

[20] González-Nicolás M.A., González-Guerrero C., Pérez-Fernández V.A., Lázaro A. (2020). Cilastatin: A potential treatment strategy against COVI-19 that may decrease viral replication and protect from the cytokine storm. Clin. Kidney J..

[21] Tejedor A., Torres A.M., Castilla M., Lazaro J.A., de Lucas C., Caramelo C. (2007). Cilastatin protection against cyclosporin A-induced nephrotoxicity: Clinical evidence. Curr. Med. Res. Opin..

[22] Carmellini M., Matteucci E., Boggi U., Cecconi S., Giampietro O., Mosca F. (1998). Imipenem/cilastatin reduces cyclosporin-induced tubular damage in kidney transplant recipients. Transplant. Proc..

[23] Markewitz A., Hammer C., Pfeiffer M., Zahn S., Drechsel J., Reichenspurner H., Reichart B. (1994). Reduction of cyclosporine induced nephrotoxicity by cilastatin following clinical heart transplantation. Transplantation.

[24] Zacharias M., Mugawar M., Herbison G.P., Walker R.J., Hovhannisyan K., Sivalingam P., Conlon N.P. (2013). Interventions for protecting renal function in the perioperative period. Cochrane Database Syst. Rev..

[25] Dagel T., Misirlioglu S., Tanju S., Afsar B., Selcukbiricik F., Erus S., Vatansever D., Balik E., Taskira C., Dilege S. (2018). Hyperthermic intraperitoneal chemotherapy is an independent risk factor for development of acute kidney injury. J BUON.

[26] Hakeam H.A., Breakiet M., Azzam A., Nadeem A., Amin T. (2014). The incidence of cisplatin nephrotoxicity post hyperthermic intraperitoneal chemotherapy (HIPEC) and cytoreductive surgery. Renal Fail.

[27] Barton C.D., Pizer B., Jones C., Oni L., Pirmohamed M., Hawcutt D.B. (2018). Identifying cisplatin-induced kidney damage in paediatric oncology patients. Pediatr. Nephrol..

[28] Zivanovic O., Abramian A., Kullmann M., Fuhrmann C., Coch C., Hoeller T., Ruehs H., Keyver-Paik M.D., Rudlowski C., Weber S. (2015). HIPEC ROC I: A phase I study of cisplatin administered as hyperthermic intraoperative intraperitoneal chemoperfusion followed by postoperative intravenous platinum-based chemotherapy in patients with platinum-sensitive recurrent epithelial ovarian cancer. Int. J. Cancer.

[29] Colantonio L., Claroni C., Fabrizi L., Marcelli M.E., Sofra M., Giannarelli D., Garofalo A., Forastiere E. (2015). A randomized trial of goal directed vs. standard fluid therapy in cytoreductive surgery with hyperthermic intraperitoneal chemotherapy. J. Gastrointest. Surg..

[30] Dickey D.T., Wu Y.J., Muldoon L.L., Neuwelt E.A. (2005). Protection against cisplatin-induced toxicities by N-acetylcysteine and sodium thiosulfate as assessed at the molecular, cellular, and in vivo levels. J. Pharmacol. Exp. Ther..

[31] Bouhadjari N., Gabato W., Calabrese D., Msika S., Keita H. (2016). Hyperthermic intraperitoneal chemotherapy with cisplatin: Amifostine prevents acute severe renal impairment. Eur. J. Surg. Oncol..

[32] Vaira M., Barone R., Aghemo B., Mioli P.R., De Simone M. (2001). Renal protection with amifostine during intraoperative peritoneal chemohyperthermia (IPCH) with cisplatin (CDDP) for peritoneal carcinosis. Phase 1 study. Minerva Med..

[33] Gruss E., Tomás J.F., Bernis C., Rodriguez F., Traver J.A., Fernández-Rañada J.M. (1996). Nephroprotective effect of cilastatin in allogeneic bone marrow transplantation. Results from a retrospective analysis. Bone Marrow Transplant..

[34] Cata J.P., Zavala A.M., Van Meter A., Williams U.U., Soliz J., Hernandez M., Owusu-Agyemang P. (2018). Identification of risk factors associated with postoperative acute kidney injury after cytoreductive surgery with hyperthermic intraperitoneal chemotherapy: A retrospective study. Int. J. Hyperth..

[35] I-Lin Sin E., Shulyn Chia C., Hwei Ching Tan G., Chee Soo K., Ching-Ching Teo M. (2017). Acute kidney injury in ovarian cancer patients undergoing cytoreductive surgery and hyperthermic intra-peritoneal chemotherapy. Int. J. Hyperth..

[36] Galfetti E., Cerutti A., Ghielmini M., Zucca E., Wannesson L. (2020). Risk factors for renal toxicity after inpatient cisplatin administration. BMC Pharmacol. Toxicol..

[37] Cascales-Campos P.A., López-López V., Muñoz-Casares F.C., Feliciangeli E., Torres Melero J., Barrios P., Morales R., Ramos I., Ortega G., Camps B. (2016). Morbidity and mortality outcomes after cytoreductive surgery and hyperthermic intraperitoneal chemotherapy in patients aged 75 years and over: Spanish group of peritoneal cancer surgery (GECOP) multicenter study. Surg. Oncol..

[38] Eng O.S., Dumitra S., O’Leary M., Raoof M., Wakabayashi M., Dellinger T.H., Han E.S., Lee S.J., Paz I.B., Lee B. (2017). Association of fluid administration with morbidity in cytoreductive surgery with hyperthermic intraperitoneal chemotherapy. JAMA Surg..

[39] Cornelison T.L., Reed E. (1993). Nephrotoxicity and hydration management for cisplatin, carboplatin, and ormaplatin. Gynecol. Oncol..

[40] Giglio M., Dalfino L., Puntillo F., Brienza N. (2019). Hemodynamic goal-directed therapy and postoperative kidney injury: An updated meta-analysis with trial sequential analysis. Crit. Care.

[41] Crona D.J., Faso A., Nishijima T.F., Mcgraw K.A., Galsky M., Milowsky M.I. (2017). A Systematic Review of Strategies to Prevent Cisplatin-Induced Nephrotoxicity. Oncologist.

[42] Bush K., Fisher J.F. (2011). Epidemiological expansion, structural studies, and clinical challenges of new β-lactamases from gram-negative bacteria. Annu. Rev. Microbiol..

[43] Hoste E.A., Clermont G., Kersten A., Venkataraman R., Angus D.C., De Bacquer D., Kellum J.A. (2006). RIFLE criteria for acute kidney injury are associated with hospital mortality in critically ill patients: A cohort analysis. Crit. Care.

[44] O’Riordan A., Wong V., McQuillan R., McCormick P.A., Hegarty J.E., Watson A.J. (2007). Acute renal disease, as defined by the RIFLE criteria, post-liver transplantation. Am. J. Transplant..

[45] Silver S.A., Harel Z., McArthur E., Nash D.M., Acedillo R., Kitchlu A., Garg A.X., Chertow G.M., Bell C.M., Wald R. (2018). Causes of Death after a Hospitalization with AKI. J. Am. Soc. Nephrol..

[46] Bucaloiu I.D., Kirchner H.L., Norfolk E.R., Hartle J.E., Perkins R.M. (2012). Increased risk of death and de novo chronic kidney disease following reversible acute kidney injury. Kidney Int..

[47] Humanes B., Jado J.C., Camaño S., López-Parra V., Torres A.M., Álvarez-Sala L.A., Cercenado E., Tejedor A., Lázaro A. (2015). Protective Effects of Cilastatin against Vancomycin-Induced Nephrotoxicity. Biomed. Res. Int..

[48] Jado J.C., Humanes B., González-Nicolás M.A., Camaño S., Lara J.M., López B., Cercenado E., García-Bordas J., Tejedor A., Lázaro A. (2020). Nephroprotective Effect of Cilastatin against Gentamicin-Induced Renal Injury In Vitro and In Vivo without Altering Its Bactericidal Efficiency. Antioxidants.

[49] Pérez M., Castilla M., Torres A.M., Lázaro J.A., Sarmiento E., Tejedor A. (2004). Inhibition of brush border dipeptidase with cilastatin reduces toxic accumulation of cyclosporin A in kidney proximal tubule epithelial cells. Nephrol. Dial. Transplant..

[50] Lazaro A., Camaño S., Humanes B., Tejedor A., Gallelli L. (2012). Novel strategies in drug-induced acute kidney injury. Pharmacology.

[51] Dimanche-Boitrel M.T., Meurette O., Rebillard A., Lacour S. (2005). Role of early plasma membrane events in chemotherapy-induced cell death. Drug Resist. Update.

[52] Holditch S.J., Brown C.N., Lombardi A.M., Nguyen K.N., Edelstein C.L. (2019). Recent Advances in Models, Mechanisms, Biomarkers, and Interventions in Cisplatin-Induced Acute Kidney Injury. Int. J. Mol. Sci..

[53] Hori Y., Aoki N., Kuwahara S., Hosojima M., Kaseda R., Goto S., Iida T., De S., Kabasawa H., Kaneko R. (2017). Megalin Blockade with Cilastatin Suppresses Drug-Induced Nephrotoxicity. J. Am. Soc. Nephrol..

[54] Lopes J.A., Jorge S. (2013). The RIFLE and AKIN classifications for acute kidney injury: A critical and comprehensive review. Clin. Kidney J..

[55] Portilla A.G., Shigeki K., Dario B., Marcello D. (2008). The intraoperative staging systems in the management of peritoneal surface malignancy. J. Surg. Oncol..

[56] González-Moreno S., Kusamura S., Baratti D., Deraco M. (2008). Postoperative residual disease evaluation in the locoregional treatment of peritoneal surface malignancy. J. Surg. Oncol..

[57] Sugarbaker P.H. (2005). Technical Handbook for the Integration of Cytoreductive Surgery and Perioperative Intraperitoneal Chemotherapy into the Surgical Management of Gastrointestinal and Gynecologic Malignancy.

[58] Cashin P.H., Ehrsson H., Wallin I., Nygren P., Mahteme H. (2013). Pharmacokinetics of cisplatin during hyperthermic intraperitoneal treatment of peritoneal carcinomatosis. Eur. J. Clin. Pharmacol..

